# Oxidative stress in coronary artery bypass surgery

**DOI:** 10.5935/1678-9741.20150052

**Published:** 2015

**Authors:** Amaury Edgardo Mont’Serrat Ávila Souza Dias, Petr Melnikov, Lourdes Zélia Zanoni Cônsolo

**Affiliations:** 1 Federal University of Mato Grosso do Sul Hélio Mandeta Medical School, Campo Grande, MS, Brazil.; 2 Moscow State Universit, Moscow, Russia.

**Keywords:** Cardiopulmonary Bypass, Oxidative Stress, Myocardial Revascularization

## Abstract

**Objective:**

The aim of this prospective study was to assess the dynamics of oxidative stress during coronary artery bypass surgery with cardiopulmonary bypass.

**Methods:**

Sixteen patients undergoing coronary artery bypass grafting were enrolled. Blood samples were collected from the systemic circulation during anesthesia induction (radial artery - A1), the systemic venous return (B1 and B2) four minutes after removal of the aortic cross-clamping, of the coronary sinus (CS1 and CS2) four minutes after removal of the aortic cross-clamping and the systemic circulation four minutes after completion of cardiopulmonary bypass (radial artery - A2). The marker of oxidative stress, malondialdehyde, was measured using spectrophotometry.

**Results:**

The mean values of malondialdehyde were (ng/dl): A1 (265.1), B1 (490.0), CS1 (527.0), B2 (599.6), CS2 (685.0) and A2 (527.2). Comparisons between A1/B1, A1/CS1, A1/B2, A1/CS2, A1/A2 were significant, with ascending values (*P*<0.05). Comparisons between the measurements of the coronary sinus and venous reservoir after the two moments of reperfusion (B1/B2 and CS1/CS2) were higher when CS2 (*P*<0.05). Despite higher values ​​after the end of cardiopulmonary bypass (A2), when compared to samples of anesthesia (A1), those show a downward trend when compared to the samples of the second moment of reperfusion (CS2) (*P*<0.05).

**Conclusion:**

The measurement of malondialdehyde shows that coronary artery bypass grafting with cardiopulmonary bypass is accompanied by increase of free radicals and this trend gradually decreases after its completion. Aortic clamping exacerbates oxidative stress but has sharper decline after reperfusion when compared to systemic metabolism. The behavior of thiobarbituric acid species indicates that oxidative stress is an inevitable pathophysiological component.

## Abstract (pt)

**Objetivo:**

O objetivo deste estudo prospectivo foi avaliar a dinâmica do estresse oxidativo durante a cirurgia de revascularização miocárdica com circulação extracorpórea.

**Métodos:**

Participaram 16 pacientes submetidos à revascularização miocárdica. As amostras de sangue foram coletadas da circulação sistêmica, no momento da indução anestésica (artéria radial - A1), do retorno venoso sistêmico (B1 e B2), quatro minutos após a remoção do pinçamento aórtico, do seio coronariano (SC1 e SC2), quatro minutos após a remoção do pinçamento aórtico, e da circulação sistêmica, quatro minutos após finalização da circulação extracorpórea (artéria radial - A2). O marcador do estresse oxidativo, malondialdeído, foi dosado utilizando espectrofotometria.

**Resultados:**

Os valores médios de malondialdeído foram (ng/dl): A1 (265,1), B1 (490,0), SC1 (527,0), B2 (599,6), SC2 (685,0) e A2 (527,2). As comparações entre A1/B1, A1/SC1, A1/B2, A1/SC2, A1/A2 foram significativas, com valores ascendentes (*P*<0,05). As comparações entre as dosagens do seio coronário e reservatório venoso após os dois momentos de reperfusão (B1/SC1 e B2/SC2) foram mais elevadas no momento SC2 (*P*<0,05). Apesar dos valores mais elevados após o término da circulação extracorpórea (A2), quando comparadas às amostras da indução anestésica (A1), aqueles apresentam tendência de queda quando comparadas as amostras do segundo momento de reperfusão (SC2) (*P*<0,05).

**Conclusão:**

As dosagens de malondialdeído mostram que a revascularização miocárdica com circulação extracorpórea é acompanhada de aumento de radicais livres com tendência deste diminuir progressivamente após seu término. O pinçamento aórtico exacerba o estresse oxidativo, porém apresenta queda mais acentuada após a reperfusão quando comparadas ao do metabolismo sistêmico. O comportamento das espécies reativas ao ácido tiobarbitúrico indica que o estresse oxidativo é um componente patofisiológico inevitável.

**Table:** 

**Abbreviations, acronyms & symbols**	
CPB	Cardiopulmonary bypass
FR	Free radicals
MDA	Malondialdehyde
TBARS	Reactive species thiobarbituric acid

## INTRODUCTION

For several decades, the myocardial ischemic syndromes are the subject of various investigations into the etiology, pathogenesis, progression and treatment in order to achieve the best outcome.

Despite the numerous clinical trials in order to shorten the ischemia and therefore limit the extent of injury, the reintroduction of oxygen to an ischemic means initiates a complex chain of events leading to additional tissue injury^[1,2]^, which do not ensure the maintenance of ventricular function.

During normal myocyte metabolism, complete reduction of an oxygen molecule in the electron transport chain, requires addition of four electrons. Due to its electronic conformation, oxygen tends to receive an electron at a time (monovalent reduction), culminating in the formation of reactive intermediates of oxygen - reactive oxygen species metabolism (ROMs), such as superoxide radicals (O^2-·^) hydroperoxyl (HO^2·^) and hydroxyl (OH^·^) and hydrogen peroxide (H_2_O_2_)^[3]^.

The reactive oxygen species are important contributors to the reperfusion injury^[4,5]^. After the onset of reperfusion there is a respiratory "explosion" by the input of O_2_ lasting for several minutes and persistently elevated superoxide production^[6,7]^.

Studies have demonstrated that the generation of free radicals (FR) is maximum in 3 to 5 minutes of reperfusion and lasting up to 3h^[8,9]^, which significantly contributes to the myocardial depression^[10,11]^.

The formation of free radicals can compromise several cell elements, causing protein denaturation, DNA chain breaks, enzymatic inactivation and lipid peroxidation^[12]^.

The damage to lipids induces lipid peroxidation. One of the most popular products of lipid peroxidation is malondialdehyde (MDA)^[13]^, which is the final product of non-enzymatic degradation of polyunsaturated fatty acids. High MDA levels increase the formation of lipoperoxide and indicate increased lipid peroxidation^[14]^.

The membranes are mainly composed of phospholipids and proteins. Changes in membrane lipids are among the main events during ischemia and reperfusion, thus losing the selectivity in ion exchange, release of hydrolytic enzymes, formation of cytotoxic products, ending with cell death^[15,16]^.

There are some methods of measurement of free radicals, among which stands out the detection of reactive species thiobarbituric acid (TBARS), which aims to detect lipid peroxidation of the cell membrane^[17]^.

We know that in cardiac surgery, the use of cardiopulmonary bypass causes global systemic ischemia, leading to exacerbate the release of free radicals. In addition, some intraoperative techniques adopt ischemia and reperfusion as myocardial protection method, either associated with various types of cardioplegic solutions or just under mild hypothermia.

Thus, the careful study of ischemia-reperfusion process is of paramount importance, since the techniques adopted during CABG are peculiar in the induction of this process, and that intermittent aortic clamping can also contribute to this mechanism.

### General Objectives

Assessing the dynamics of concentrations of thiobarbituric acid reactive substances during coronary artery bypass grafting with cardiopulmonary bypass and intermittent aortic clamping.

### Specific objectives

Determining the concentrations of thiobarbituric acid reactive substances from the systemic venous return during cardiopulmonary bypass, and venous return from the coronary sinus after myocardial reperfusion.

Assessing the dynamics of dosages of thiobarbituric acid reactive substances during the procedure.

Comparing the dynamic element studied between assays from the systemic circulation and the coronary sinus.

## METHODS

The study was approved by the Medical Ethics Committee of the Universidade Federal do Mato Grosso do Sul (Protocol 1926) and the written informed consent form was signed by each patient participating in the study.

It was a prospective study performed at the Cardiovascular Surgery Service of the University Hospital of the Universidade Federal do Mato Grosso do Sul.

### Population

The study included sixteen patients, 5 females and 11 males, who underwent coronary artery bypass grafting with cardiopulmonary bypass for making 2 or more grafts.

Patients with acute or chronic renal failure, diabetes mellitus, heart rate in atrial fibrillation, patients with dilated cardiomyopathy or associated valvular lesions, patients undergoing emergency surgery or patients using any medication suppressing free radicals as vitamin C, n-acetylcysteine, allopurinol, immunomodulatory or corticosteroids not were included.

### Cardiopulmonary bypass

After pattern longitudinal median sternotomy, for cardiopulmonary bypass (CPB) a cannula in the ascending aorta was installed and double venous and coronary sinus drainage were performed in order to collect selective samples. The CPB was performed in mild hypothermia (32°C) and hemodilution. The technique for revascularization was intermittent aortic cross-clamping, adopted as routine by the Cardiovascular Surgery Service of the University Hospital of the Universidade Federal do Mato Grosso do Sul. Roller pump in the arterial line and hollow fiber membranes oxygenators with arterial line filter (Braile Biomédica - São José do Rio Preto - SP) were used and priming calculated as the perfusion hematocrit – 20 mg/dl.

The electrolyte and metabolic balance was maintained in accordance with the metabolic needs of the patient during standard procedure. The perfusion technique adopted was alpha pH stat.

Anesthesia was performed according to established protocols, but the use of possible free radical suppressor as ascorbic acid and n-acetylcysteine were not used.

### Blood and laboratory analysis

Moments of collection were:

A1 - blood collected at time of anesthetic induction - sample of the radial artery.

A2 - blood collected four minutes after completion of CPB - sample of the radial artery.

B1 - blood collected from the venous line of the cava after 4 minutes of the first unclamping.

B2 - blood collected from the venous line of the cava after 4 minutes of the second unclamping.

CS1 - blood from the venous return of the coronary sinus after 4 minutes of the first unclamping.

CS2 - blood collected from the coronary sinus venous return after 4 minutes of the second unclamping.

Blood samples were collected in polypropylene syringes and immediately transferred to vacuum tubes (BD Vacutainer Systems, Becton, Dickinson & Co). Serum was separated by centrifugation (3.000×g, 15 min), and transferred to demineralized Eppendorf tubes, and stored at -18°C for later determination of malondialdehyde. All materials, plastics or glasses were immersed for 24 hours in 5% Extran solution (Merck), rinsed and immersed for at least 24 hours in a 10% ultrapure solution of nitric acid (Merck) for waste decontamination. Then, they were washed with ultrapure water (Milli-Q, Millipore, Bedford, USA) and dried at 40°C.

The plasmatic concentrations of the substances that react with thiobarbituric acid (TBARS) were measured by spectrophotometric method. The test used to evaluate cellular damage by lipid peroxidation is based on the study by Percário et al.^[17]^. The volume of 1 ml TBA (thiobarbituric acid) (10 nM/l) was added to 0.5 ml of the sample. A standard solution consisting of 1 ml TBA (10 nM/l) and 0.5 ml of MDA was prepared (20 nM/L). A third solution containing 1 ml of TBA (10 nM/l) and 0.5 ml of water, served as background reading of the spectrophotometer. These solutions were heated in water bath at 94°C for 1 hour and then cooled in running water for 5 minutes. To block reaction we added 4 ml of n-butyric alcohol in each tube. The tubes were vortexed for complete extraction of the MDA into the organic phase of the system, then centrifuged at 2500 rpm for 10 minutes. At this time, there is phase separation, and 3 ml of the organic phase (surface) was aspirated for spectrophotometer reading.

The reading was performed at 532 nm. The final amount of MDA in ng/dl is obtained by using the following formula:

MDA = A average x F, where A average = (A1+A2)/2. F=4406.1/A Standard MDA, where A is absorbance.

### Statistical analysis

Comparisons of measurements of TBARS in the times of aortic clamping in the radial artery, venous return and coronary sinus were performed with the Student t test for paired data at the 95% level of significance.

The tests involving correlation between variables were performed via linear correlation coefficient of Pearson and its respective t-Student test.

The normality of the data, via criteria of Kolmogorov-Smirnov also at the 95% level of significance was tested.

## RESULTS

This study evaluated 16 patients being 5 females (31.2%) and 11 males (68.8%) with mean age of 60±8.6 years (mean±SD).

The ventricular ejection fraction prior to surgery was 51.6±9.7. The total time of cardiopulmonary bypass was 68.1±23.2 min. The average times of the 1^st^ and 2^nd^ aortic clamping were 9.9±2.0 min and 9.9±2.5 min, respectively (Table 1).

**Table 1:** Ejection fraction (%), aortic clamping time and CPB time.

Parameters	Mean ± SD
Ejection fraction	38.0 - 70.0 (51.6±9.7)
1^st^ Clamping time (min)	7.0 - 13.0 (9.9±2)
2^nd^ Clamping time (min)	7.0 - 15.0 (9.9±2.5)
CPB time (min)	40.0 - 120.0 (68.1±23.2)

CPB=cardiopulmonary bypass

The data in Table 2 show the analysis comparing each individual from the anesthetic induction and after the first aortic unclamping (4 min reperfusion), or induction of anesthesia/venous line (A1/B1) and induction of anesthesia/Coronary sinus (A1/CS1). In the table are shown the average values for each site, the average difference found and evaluation of significance by Student's t test for paired data.

**Table 2:** Comparison of the dosages of TBARS (ng/ml) between the times A1 - B1 and A1 - CS1*.

	Time	Mean ± SD	*P*
TBARS	A1	265.1±233.5	<0.001
	B1	490±360.9	
	A1	265.1±233.5	<0.001
	CS1	527±358.7	

* A1=anesthetic induction; B1=systemic venous return, four minutes after the first unclamping; CS1=coronary sinus, four minutes after the first unclamping; TBARS=reactive species thiobarbituric acid

Table 3 shows the comparisons between assays of TBARS during anesthetic induction and the venous line and coronary sinus with 4 min of reperfusion after the 2^nd^ aortic unclamping, or that is, A1/B2 and A1/CS2. The analysis was performed in each patient, using the Student t test for paired data.

**Table 3:** Comparison of the dosages of TBARS (ng/ml) between the times A1 - B2 and A1 - CS2*.

	Time	Mean ± SD	*P*
TBARS	A1	265.1±233.5	<0.001
	B2	599.6±346.7	
	A1	265.1±233.5	<0.001
	CS2	684.9±386.7	

* A1=anesthetic induction; B2=systemic venous return, four minutes after the second unclamping; CS2=coronary sinus, four minutes after the second unclamping; TBARS=reactive species thiobarbituric acid

Comparisons of TBARS levels between the two periods of reperfusion, or that is, between samples collected in the venous line and coronary sinus after 4 min the removal of each of the two aortic clamping performed (B1/B2 and CS1/CS2) are presented in Table 4. The analysis was performed in each patient, using the Student t test for paired data.

**Table 4:** Comparison of the dosages of TBARS (ng/ml) between the moments B1 - B2 and CS1 - CS2*.

	Time	Mean ± SD	*P*
TBARS	B1	490±360.9	0.008
	B2	599.6±346.7	
	CS1	527±358.7	<0.001
	CS2	684.9±386.7	

* B1=systemic venous return, four minutes after the first unclamping; CS1=coronary sinus, four minutes after the first unclamping; B2=systemic venous return, four minutes after the second unclamping; CS2=coronary sinus, four minutes after the second unclamping; TBARS=reactive species thiobarbituric acid

Table 5 presents the comparisons of TBARS levels between the last period of reperfusion and after the end of cardiopulmonary bypass, or that is, B2/A2 and CS2/A2. The analysis was performed in each patient, using the Student t test for paired data.

**Table 5:** Comparison of the dosages of TBARS (ng/ml) between the moments B2 - A2 and CS2 - A2*

	Time	Mean ± SD	*P*
TBARS	B2	599.6±346.7	0.104
	A2	494.5±336.7	
	CS2	684.9±386.7	0.007
	A2	494.5±336.7	

* *B2=systemic venous return, four minutes after the second unclamping; CS2=coronary sinus, four minutes after the second unclamping; A2=4 minutes after completion of CPB; TBARS=reactive species thiobarbituric acid

Table 6 presents the comparisons of TBARS levels between the venous line and the coronary sinus, after the two moments of reperfusion collected after 4 minutes of each unclamping, or that is, B1/CS1, B2/CS2. The analysis was performed in each patient, using the Student t test for paired data.

**Table 6:** Comparison of dosages of TBARS (ng/ml) between the moments B1 - CS1 and B2 - CS2 *.

	Unclamping	Time	Mean ± SD	*P*
TBARS	1^st^	B1	490±360.9	0.10
		CS1	527±358.7	
	2^nd^	B2	599.6±346.7	0.03
		CS2	684.9±386.7	

* B1=systemic venous return, four minutes after the first unclamping; CS1=coronary sinus, four minutes after the first unclamping; B2=systemic venous return, four minutes after the second unclamping; CS2=coronary sinus, four minutes after the second unclamping; A2=4 minutes after completion of cardiopulmonary bypass; TBARS=reactive species thiobarbituric acid

Table 7 compares the dosages of TBARS between anesthetic induction and after the end of cardiopulmonary bypass, or that is, A1/A2. The analysis was performed in each patient, using the Student t test for paired data.

**Table 7:** Comparison of dosages of TBARS (ng/ml) between the moments A1- A2*.

Element	Time	Mean ± SD	*P*
TBARS	A1	265.1±233.5	<0.001
	A2	494.5±336.7	

* A1=anesthetic induction; A2=4 minutes after completion of cardiopulmonary bypass; TBARS=reactive species thiobarbituric acid

## DISCUSSION

During cardiac surgery with cardiopulmonary bypass, a series of inflammatory and immunological changes occur triggering oxidative stress. In non-physiological conditions during CPB, due to changes related to ischemia and reperfusion, there is an increase in free radicals and ROMs. In myocardial ischemic syndromes, it is well established that oxygen reintroduced in the myocardium during reperfusion causes significant injury. This stress is one of the initiators of this resulting myocardial damage^[1,2]^.

Patients who met the criteria for this study were suffering from acute coronary syndrome with classic indications of myocardial revascularization. The time interval between onset of symptoms of acute coronary syndrome, or that is, from the diagnosis until the surgical treatment was 7 to 10 days. This period corresponds to the time required to optimize the treatment and stabilization of the clinical condition of patients.

Our study has shown the prevalence of male patients similar to statistical indices established both at global and national levels. The age group also does not contradict the data already known from the literature.

The CPB used during cardiac surgery triggers an inflammatory reaction and consequent oxidative stress that is directly related to its duration^[18]^. In the present study, the mean CPB time was 68 minutes consistent with other studies^[18-20]^. Three patients required CPB time greater than 90 min due to the need for more grafts. However there was no correlation with perioperative complications.

It is known that the inflammatory response related to the CPB is time dependent and that is closely related to oxidative stress, which could result in high oxidative stress measurements. Despite this assumption, this study and others published in the literature^[18-20]^, since they have lower CPB time of 120 min, there was no evidence positive relationship between CPB time and oxidative stress.

The metabolic parameters measured during the entire surgical procedure remained within limits compatible with the intraoperative stability.

The reintroduction of blood in the coronary circulation after ischemia period produced by CPB and aortic clamping can lead to oxidative stress formation induced by oxygen free radicals and other ROMs, which can result in cellular injury.

Ragab et al.^[21]^ evaluated a total of 65 patients with unstable angina and acute myocardial infarction, determined the MDA levels in the acute phase of the syndrome and found high values with respect to the control group.

**Fig. 1:**
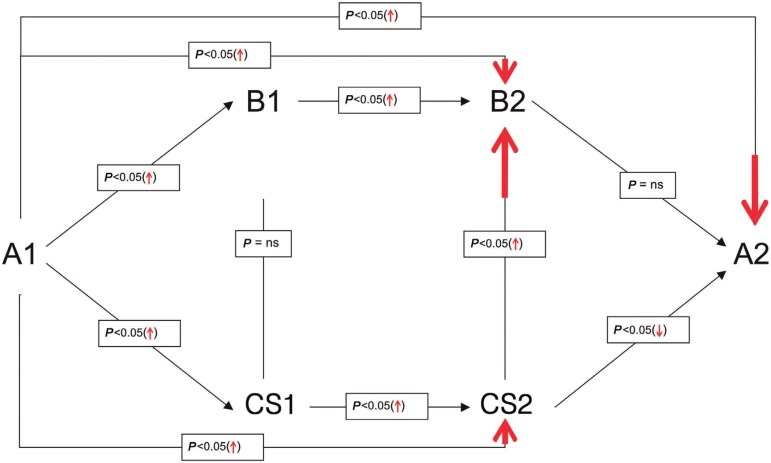
Schematic representation of the dynamics of TBARS concentrations. (↑ ↓) means the variation signal.

Patients in the present study had normal levels of MDA (265.1 ng/ml) at time A1 (Figure 1), indicating that the previous acute ischemic event has been alleviated. Thus, the stabilization period adopted in the clinical treatment of the patient is essential for mitigation of the oxidative stress produced.

It has been shown that patients undergoing CABG have no increase in TBARS between anesthetic induction and initiation of CPB. This proves by itself, that the surgical trauma is not responsible for the increased oxidative stress^[19,22,23]^. This observation enabled this study, neglecting the analytical dosage between the two mentioned moments.

In this study, the overall results were similar to the literature data known beforehand. A comparison of assays performed in the moments A1/B1 and A1/CS1 (Figure 1) allows discovering a significant increase in TBARS levels. This can be considered as an indication that cardiopulmonary bypass is, in fact, an activation inductor of oxidative stress.

Recent literature includes data regarding the relationship between oxidative stress, lipid peroxidation and ultrastructural myocardial damage in patients with chronic coronary artery disease undergoing coronary artery bypass surgery. Thus, after the restoration of coronary perfusion, Milei et al.^[24]^ performed the determinations of oxidative stress and ultrastructural injury. The authors evaluated the TBARS production in the 5^th^ and 20^th^ min. after aortic unclamping, both from the coronary sinus effluent as the systemic circulation. It was observed that, in the coronary sinus and the systemic circulation, the TBARS concentrations were similar in both moments, concluding that the isolated myocardial production of oxidizing substances was not greater than the systemic circulation.

It is noteworthy that the first of those moments roughly corresponds to the B1 moment of our study, even if they have different aortic clamping times. Although the technique adopted was not exactly the same, the results of determination of TBARS are in agreement with the data from other methodologies.

As follows from the results of this study, in the comparison between B1 and CS1, there are no significant differences in plasma TBARS levels of both the venous blood line as the coronary sinus collected after the first unclamping. This clearly demonstrates that cross-clamping does not increase the systemic oxidative stress in addition to the already installed.

Akila et al.^[18]^ performed a study comparing coronary artery bypass grafting with and without cardiopulmonary bypass in patients not infarcted and with preserved ventricular function. The average time of myocardial ischemia in the "on pump" group was 44.4 min and the CPB time was 65.6 min. It was concluded that in patients undergoing cardiopulmonary bypass, oxidative stress is higher in this group after 15 and 60 min, returning to levels similar to "off pump" patients 24 hours after aortic unclamping. Despite not having been the objective of Akila et al.^[18]^, by thoroughly analyzing their results, we can extrapolate the data in the published tables. This allows noting that in the "on pump" subgroup, the 5^th^ min of reperfusion does not present significant increase in TBARS levels, however the increase was progressive. This becomes more evident in the 15^th^ and 60^th^ min, always presenting ascending pattern.

This deviates from our study, since it is observed a significant increase in TBARS levels after 4 min of aortic unclamping, both in the systemic circulation (venous line), and the coronary sinus (coronary circulation). In addition, oxidative stress is accentuated in the second moment of reperfusion and presents downward trend after 4 min of the completion of cardiopulmonary bypass (A2) (Figure 1). These differences may be due to the fact that, in the study by Akila et al.^[18]^, the average time of myocardial ischemia was approximately 45 min, while in this study it was approximately 20 min. Thus, a longer ischemic time would entail a tendency to perpetuate the oxidative stress and therefore progressively higher levels of TBARS. However, this would tend to return to baseline levels after 24 hours of aortic unclamping^[18]^.

In this study the extent of TBARS after 24 hours was not measured, since the aim was to compare coronary reperfusion with systemic reperfusion, where maintaining another catheter in the coronary sinus allocated would bring risk due to the greater invasiveness of the patient. However, there was a downward trend in TBARS concentrations at the time A2, which probably remained as the triggering factors of oxidative stress had been removed. It is possible that brief episodes of ischemia-reperfusion imposed by intermittent aortic clamping have promoted a myocardial preconditioning, thereby allowing a higher tolerance to oxidative stress, and shortening the time of TBARS levels reduction^[25]^.

Matata et al.^[19]^ compared in their study two groups of patients undergoing CABG, the first off-pump and the second with normothermic CPB. The mean CPB time was 69min and the myocardial ischemia time of 34.2 and 35.6 min in groups 1 and 2 respectively. The authors demonstrated that, in patients undergoing CPB, the MDA dosages showed ascending pattern, peaking in the fourth hour after completion of the bypass.

In the present study we obtained a bypass time, under hypothermia, similar to previous study (68.1 min.), but with a smaller myocardial ischemia time (19.8 min.). Oxidative stress measured by MDA in the systemic venous return presents ascending pattern between the first and second aortic cross-clamping and without significant reduction until the time A2 (Figure 1). In this study the TBARS levels were not measured after four hours of completion of the bypass, but the downward trend in absolute terms allows us to detect standardization, and not the perpetuation of the ascending pattern as in the study of Matata et al.^[19]^. When we compare the CS2 and A2 times (Figure 1), we confirmed the downward trend (*P*<0.05). These differences may be due to the fact that mild hypothermia, as the systemic metabolism attenuation method, may decrease the degree of damage caused by ischemia-reperfusion. Therefore, the use of this technique necessarily affects the early reduction of the levels of malondialdehyde.

Regarding the comparisons between the aortic clamping and measured levels of MDA, we observed a greater increase after the second unclamping, especially in the sample of the coronary sinus (CS2) (Figure 1). On the other hand, these concentrations gradually reduce after completion of CPB. This becomes more evident when we compare the TBARS values between CS2 and A2 times, making suppose that the intrinsic myocardial antioxidant mechanisms are more effective in attenuating oxidative stress.

Another aspect to be highlighted is the fact that at that time (A2), the completion of CABG allowed restoration of myocardial blood flow before committed by coronary artery disease. The returned circulating therein, allowed mobilizing the ischemic myocardial tissue of all organic antioxidant defenses produced, enabling an early reduction in levels of malondialdehyde.

Thus, we note that the myocardial protection methods still deserve extensive discussion, since in most studies that address the ischemia and reperfusion process, it is used as methodology the dosage of injury and myocardial necrosis markers, or that is, restricted only to hypoxia times. Thus, occurring or not the injury mediated by ischemia, reperfusion will trigger additional injury to the already installed, causing serious cardiac performance changes, often irreversible. In addition, we can also state that the investigations in the field of molecular and atomic biology aim to unravel the metabolic processes in their microstructure, in order to discover methods or subcellular mechanisms allowing them to block or mitigate the myocyte injuries. The lesion detection methodology mediated by reperfusion (TBARS) is still a field to be explored by cardiology. The direct measurement of free radicals is not a feasible procedure because the average extremely short lifetime of these substances. The methodologies used once, such as the measurement of methylene blue does not allow to obtain reliable data due to partial absorption of this compound, which leads to serious errors in determining these radicals. The instrumental methods such as electron paramagnetic resonance are not readily available for this purpose.

The method using the measurement of thiobarbituric acid reactive species (TBARS), which was used in this study, is an approximation of the ideal method. However, it is known that the malondialdehyde (MDA) - destruction product of the compounds containing unpaired electrons – is not only the indicator of oxidative stress. It is known that it is only a window on the Universe from free radicals. Nevertheless, for purposes of comparison with the data available in the literature, this approach is acceptable.

The intermittent aortic clamping technique is little adopted in the studies presented in the literature. Nevertheless, comparisons with other methods show that intermittent aortic clamping can shorten the lesions mediated by reperfusion. Notwithstanding this, we also evidenced that although the various methods of myocardial protection, oxidative stress always occur. Thus, it is up to the cardiovascular surgeon to investigate new ways, devices or medications that enable the optimization of organic patient's own defenses against oxidative stress.

Thus, it opens up a vast field of research based on molecular and atomic biology, aiming to reduce the injuries of binomial ischemia-reperfusion.

## CONCLUSION

The results of malondialdehyde dosages clearly show that myocardial revascularization with cardiopulmonary bypass is accompanied by elevation of free radicals with this trend to gradually decrease after its completion. The aortic clamping exacerbates the oxidative stress of the coronary sinus venous return when compared to dosages of systemic venous return, but the measurements of coronary effluent presents sharpest decline after reperfusion compared to the systemic metabolism. The behavior of thiobarbituric acid reactive species, despite differences in methodologies, presents similar, indicating that oxidative stress is an unavoidable pathophysiological component.

**Table:** 

**Authors’ roles & responsibilities**	
AEMSASD	Analysis and/or interpretation of the data; conception and design; implementation of projects and/or experiments; manuscript writing or critical review of its content
PM	Analysis and/or interpretation of the data; final approval of the manuscript
LZZC	Analysis and/or interpretation of the data; final approval of the manuscript
